# Characterization of alternative splicing during mammalian brain development reveals the extent of isoform diversity and potential effects on protein structural changes

**DOI:** 10.1242/bio.061721

**Published:** 2024-10-10

**Authors:** Leila Haj Abdullah Alieh, Beatriz Cardoso de Toledo, Anna Hadarovich, Agnes Toth-Petroczy, Federico Calegari

**Affiliations:** ^1^ CRTD-Center for Regenerative Therapies Dresden, School of Medicine, TU Dresden, Germany; ^2^ Max Planck Institute of Molecular Cell Biology and Genetics, 01307 Dresden, Germany; ^3^ Center for Systems Biology Dresden, 01307 Dresden, Germany; ^4^ Cluster of Excellence Physics of Life, TU Dresden, 01062 Dresden, Germany

**Keywords:** Alternative splicing, Brain development, Neurogenesis, Protein structure prediction, Isoform-specific protein conformations

## Abstract

Regulation of gene expression is critical for fate commitment of stem and progenitor cells during tissue formation. In the context of mammalian brain development, a plethora of studies have described how changes in the expression of individual genes characterize cell types across ontogeny and phylogeny. However, little attention has been paid to the fact that different transcripts can arise from any given gene through alternative splicing (AS). Considered a key mechanism expanding transcriptome diversity during evolution, assessing the full potential of AS on isoform diversity and protein function has been notoriously difficult. Here, we capitalize on the use of a validated reporter mouse line to isolate neural stem cells, neurogenic progenitors and neurons during corticogenesis and combine the use of short- and long-read sequencing to reconstruct the full transcriptome diversity characterizing neurogenic commitment. Extending available transcriptional profiles of the mammalian brain by nearly 50,000 new isoforms, we found that neurogenic commitment is characterized by a progressive increase in exon inclusion resulting in the profound remodeling of the transcriptional profile of specific cortical cell types. Most importantly, we computationally infer the biological significance of AS on protein structure by using AlphaFold2, revealing how radical protein conformational changes can arise from subtle changes in isoforms sequence. Together, our study reveals that AS has a greater potential to impact protein diversity and function than previously thought, independently from changes in gene expression.

## INTRODUCTION

The lack of correlation between number of genes and complexity of eukaryotic organisms has puzzled biologists for decades (Hahn and Wray, 2002; Thomas, 1971). As one example, the worm *Caenorhabditis elegans* shares a comparable number of protein-coding genes with humans, despite major differences in tissue diversity and organ complexity (Cunningham et al., 2022). The reasons for such a lack of correlation are not entirely clear but an evolutionary increase in abundance and complexity of alternative splicing (AS) can partly explain this discrepancy. In fact, changes in AS, transcriptional initiation and 3′ cleavage/polyadenylation sites have great potential to increase transcripts' diversity from a common pool of genes and, thus, considerably expand the genomic coding potential (Nilsen and Graveley, 2010; Reixachs-Solé and Eyras, 2022). Consistently, frequency and heterogeneity of AS have expanded across evolution (Schaefke et al., 2018; Verta and Jacobs, 2022) and positively correlate with organism complexity (Chen et al., 2014; Yang et al., 2021). Although its full extent and biological significance remain poorly investigated (Pickrell et al., 2010; Saudemont et al., 2017; Tress et al., 2017), AS is thought to have enormous potential to redefine proteins’ sequences and, as a result, modulate their function by changing their stability, localization and interaction with other molecules (Kelemen et al., 2013; Marasco and Kornblihtt, 2023).

AS profiles that are highly specific not only across species but also across organs within the same species result from the concerted modulation of AS events (i.e. inclusion/exclusion of exons, parts of exons or intron retention) (Barbosa-Morais et al., 2012; Buljan et al., 2012; Ellis et al., 2012; Rodriguez and Pozo, 2020). Notably, of different organs, the brain is among those with the highest proportion of spliced genes (Mazin et al., 2021; Yeo et al., 2004) and with highly specific and conserved AS profiles, including microexons (≤27 nt) (Irimia et al., 2014; Raj and Blencowe, 2015). One potential reason for the prevalence of AS in the brain is the extreme diversity of cell types characterizing this organ, which arises during development from neural stem cells (NSC). More specifically, during embryonic development NSC initially undergo proliferative division to expand their pool and later switch to differentiative division to generate neurogenic progenitors (NP) giving rise to neurons (N) specifying into hundreds neuronal subtypes (Villalba et al., 2021). While several studies have investigated the role of AS in the context of neuronal maturation and specification (Furlanis and Scheiffele, 2018; Raj and Blencowe, 2015; Weyn-Vanhentenryck et al., 2018; Zhang et al., 2016) and linked neural-specific splicing factors to neurodevelopmental disorders (Chau et al., 2021; Quesnel-Vallières et al., 2016), relatively little attention has focused on the role of AS in neurogenic commitment and, specifically, cell fate transition from NSC to NP. Limited examples among these studies include the identification of neurogenic-specific splicing factors (Han et al., 2022; Verdile et al., 2023), splice site mutations arising in evolution resulting in progenitor expansion (Florio et al., 2016) and crosstalk between epigenetic and splicing programs (Sahu et al., 2022). Despite of this, comprehensive annotation of isoform diversity across neuronal cell types and assessment of potential effects on protein structure are missing.

Several technical limitations contribute to making the study of AS in the context of cell fate commitment particularly challenging. For example, assessment of AS traditionally relies on annotation databases that are often neither organ- nor cell-specific and usually neglect underrepresented cell sub-populations (Morillon and Gautheret, 2019; Zhang et al., 2020). The advent of long read sequencing (LRS) has significantly improved the identification of full-length and new transcripts including novel exons and cryptic splice sites (An et al., 2018; Oikonomopoulos et al., 2020) but a reliable assessment of isoform abundance and relative proportion of splice variants remains unfeasible with this method (Sarantopoulou et al., 2021).

Conversely, quantification of relative exon inclusion relies on the use of short read sequencing (SRS) that, on the other hand, does not allow the reconstruction of full-isoform variants. Independently from the use of LRS or SRS, additional technical aspects are to be considered in the use of single-cell sequencing data to assess exon inclusion, such as, among others, biases introduced by PCR amplification, degree of RNA coverage, dropouts rates and complexity of computational analysis (Arzalluz-Luque and Conesa, 2018; Buen Abad Najar et al., 2020; Westoby et al., 2020). While great effort is being made to overcome these limitations, capturing cell type-specific AS dynamics that are both quantitative and comprehensive of full-length transcript information currently requires a combination of both SRS and LRS performed in parallel on the same cell pool. This has seldom been attempted (Glinos et al., 2022; Gupta et al., 2018; Joglekar et al., 2021; Leung et al., 2021; Qiao et al., 2020) and, to the best of our knowledge, never for specific cell types of the developing mammalian brain. It is even more limiting that systematic assessment of the consequences of AS on protein structure and putative function in cell fate commitment is entirely lacking.

To assess AS dynamics and explore its significance during brain development, we took advantage of an extensively characterized double-reporter mouse model that allows the identification of NSC, NP and N during corticogenesis by their combinatorial expression of RFP and/or GFP (Aprea et al., 2013; 2015). This dual-reporter mouse line was instrumental for the characterization of the transcriptome and epigenome defining neurogenic commitment including the functional assessment of pioneer transcription factors, different classes of non-coding RNAs (long/circular/micro) and DNA methylation (Aprea et al., 2013; 2015; Artegiani et al., 2015; Dori et al., 2019; 2020; Mestres and Calegari, 2023; Noack et al., 2019). Combining the use of this dual-reporter mouse line with LRS, SRS and bioinformatic tools, we here aimed to provide a novel workflow and resource to reconstruct cell type-specific transcriptome diversity during brain development and quantitatively assess AS events. By this, we describe nearly 50,000 new transcripts including novel exons, splice sites and/or microexons, thus uncovering the full spectrum of splicing dynamics accompanying fate transitions from NSC to NP and N.

Next, by using AlphaFold2 (Jumper et al., 2021; Varadi et al., 2022) we inferred the biological significance of several observed AS events on the resulting protein sequence and structure by modeling the 3D conformational changes resulting from isoform switching characterizing specific cell states. Remarkably, this highlighted that nearly 40% of isoform pairs originating from the same gene exhibited large global conformational changes including fold switches. In addition, we describe the occurrence of regions with nearly identical sequences adopting profoundly different secondary structures (secondary structure element switches), such as alpha-helix versus beta-sheet, depending on distant AS events, revealing that even negligible changes in exon usage can induce large conformational changes likely influencing the functional properties of proteins.

Overall, our study combines the use of a validated mouse model with new sequencing annotation, computational pipelines and advanced machine learning-based protein modeling in order to provide a comprehensive resource for the assessment of cell-type specific AS profiles and its potential biological significance in mammalian brain development.

## RESULTS

### A new cell type-specific transcriptome assembly of the developmental mouse cortex

**Fig. 1.**
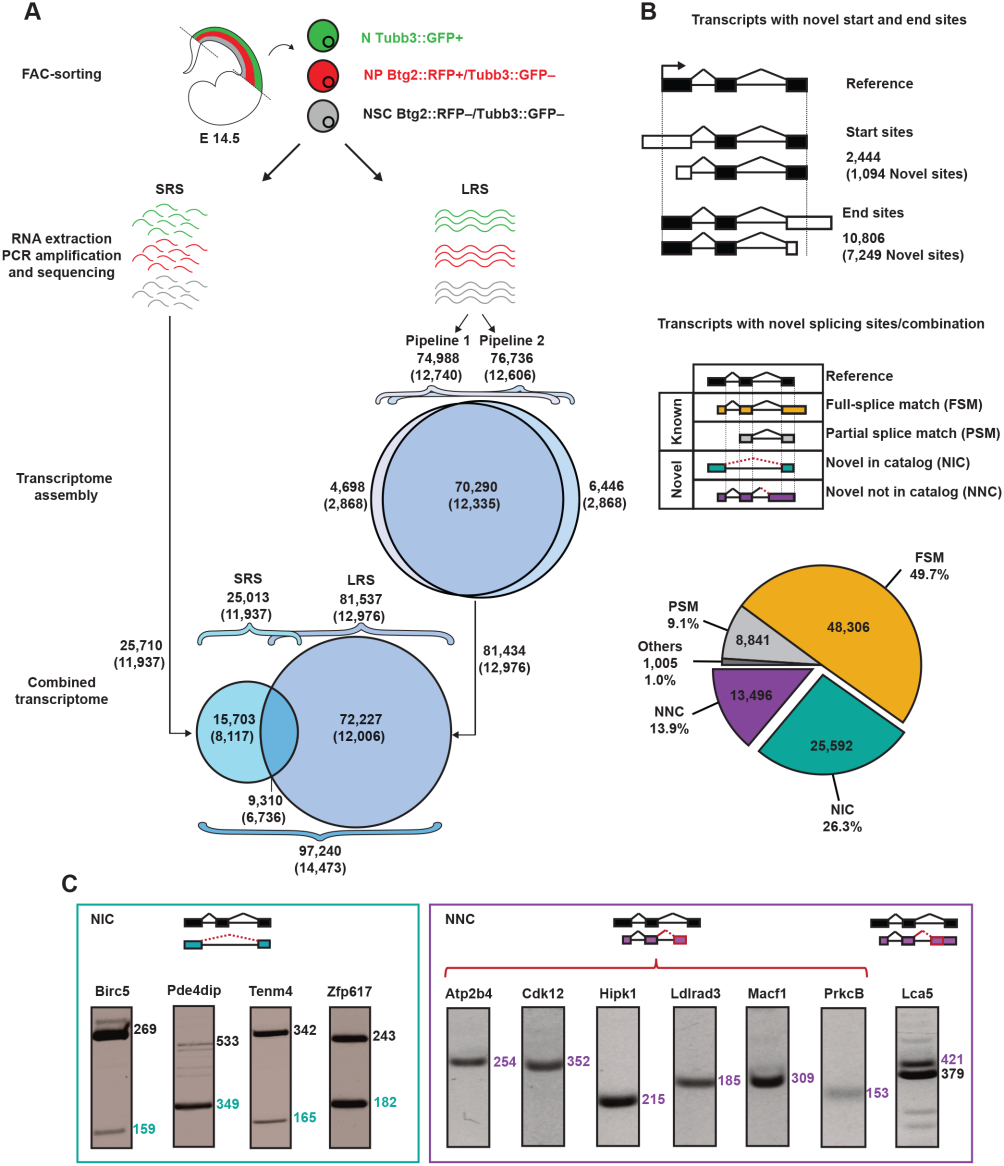
**Transcriptome assembly of NSC, NP and N.** (A) Drawing exemplifying the combination of SRS and LRS datasets from cortical cell types obtained from E14.5 Btg2::RFP/Tubb3::GFP mouse embryos as previously described (Aprea et al., 2013, 2015). Upon SRS and LRS (left and right, respectively) assembling of transcripts (genes within parentheses) was performed using Hisat2-Stringtie (SRS) or two alternative pipelines (LRS) that were merged into a combined assembly of 97,240 transcripts from 14,473 genes. For the SRS dataset, reads from several embryos pulled from three biological replicates were processed separately for each cell type and replicate and the resulting transcriptomes were merged into a single SRS assembly. For LRS, all analyses were performed on a single technical replicate derived by the pooling of NSC, NP and N obtained from lateral cortices of several embryos from three dams. (B) Classification of known and novel transcripts upon splice junction assembly obtained comparing either transcripts start/end sites (top) or internal splice junction (bottom). The latter included known transcripts whose splice junctions (continuous lines) either completely or partially matched reference junctions (full- or partial-splice match, FSM or PMS, yellow or gray, respectively), and novel transcripts whose splice junctions (dotted lines) either matched novel combinations of known junctions (novel in catalog, NIC, green) or were never described before (novel not in catalog, NNC, purple). (C) Validation of novel splice junctions (redAQ: A) resulting in one RT-PCR product when involving a novel first or last exon and in two bands when involving a novel internal exon (i.e. in Lca5).

We began our study by reconstructing a new cell type-specific transcriptome of the developing mouse cortex (Fig. 1; Fig. S1). To this end, we assembled previously generated SRS data of NSC, NP and N at the peak of neurogenesis at mouse embryonic day (E) 14.5 (Aprea et al., 2013; 2015) obtained by pulling embryos from three pregnant mice as independent biological replicates. Using Hisat2-Stringtie (Kim et al., 2019; Pertea et al., 2015), this resulted in 25,710 transcripts from 11,937 genes (Fig. 1A; left). The ratio of about 2:1 transcripts-to-genes was relatively low considering the 5:1 ratio previously reported in mouse (Cunningham et al., 2022) and likely resulted from intrinsic limitations in the reconstruction of full-length transcripts from SRS rarely spanning across multiple exons and often failing to resolve ambiguities at complex loci (Shumate et al., 2022).

Given these limitations of SRS, we next exploited the use of LRS to expand our assessment of transcriptome diversity. To this end, we extracted and sequenced in a single technical replicate mRNA from NSC, NP and N from E14.5 mouse cortices pulled together from three pregnant mice, isolated as previously described (Fig. 1A; top) (Aprea et al., 2013; 2015). PacBio high-quality transcripts (accuracy ≥99%) were next processed by two alternative bioinformatic pipelines (pipelines 1 and 2, see Materials and Methods) for quality control and removal of redundant or false-positive transcripts and artifacts. This resulted in 74,988 and 76,736 transcripts (12,740 and 12,606 genes) with about 90% overlap in both pipelines (70,290) and 4698 and 6446 transcripts being only detected by pipeline 1 or 2, respectively (Fig. 1A; right). Importantly, the vast majority of 5′ (>85%) and 3′ (>95%) ends of these pipeline-specific transcripts were supported by Ensembl coordinates, data from CAGE and poly(A) motifs and/or previous reports (Veiga et al., 2022) and therefore unlikely to be the product of degradation (data not shown). For these reasons, we decided to merge all LRS data into a unique assembly of 81,434 transcripts (12,976 genes) and further combine these with the 25,710 transcripts predicted from SRS data. After merging (see Materials and Methods and Fig. S1A and B), we obtained a novel transcriptome assembly of 97,240 transcripts (14,473 genes), with 15,703 and 72,227 transcripts deriving from only SRS and LRS datasets, respectively, and 9,310 from both assemblies (Fig. 1A; bottom). With a new transcripts-to-genes approaching the 7:1 ratio, this exceeded by almost 2-fold the magnitude of transcript diversity recently described in the adult brain (Leung et al., 2021). This is remarkable when considering that the E14.5 mouse brain is expected to contain a much lesser degree of cell diversity than the adult brain.

In addition to 2444 and 10,806 transcripts with novel transcription start (1094) or end (7249) site, respectively, we found that 40% of transcripts in our assembly were not annotated in any database and were therefore novel either due to the presence of new splice sites (novel not in catalog, NNC) or splice sites that are known but combined in new ways (novel in catalog, NIC) (13,496 and 25,592 transcripts from 5392 and 6635 genes, i.e. ∼14% and ∼26%, respectively) (Fig. 1B). Novel isoforms were detected in SRS and/or LRS assemblies, with several being supported by both sequencing approaches (Fig. S2).

We then sought to validate the existence of novel transcripts belonging to both categories. We selected candidate novel splice junctions by several criteria including: (i) coverage by ≥30 junction reads, (ii) low variability among replicates (coefficient of variation<0.5), (iii) unique new junction per gene, and (iv) belonging to the top 85% most expressed genes. This resulted in a subset of 471 candidates of which only about 140 (30%) were predicted to give a maximum of 2 PCR products and, thus, be suitable candidates for validation by RT-PCR. Among these 140, we finally selected 11 for validation, which was successful in all cases (Fig. 1C and Fig. S2).

Taken together, our analysis provides the most complete cell type-specific assembly of full-length transcriptomes of the developing mammalian cortex to date including nearly 50,000 potentially new transcripts.

### Patterns of AS characterizing neurogenic commitment

**Fig. 2.**
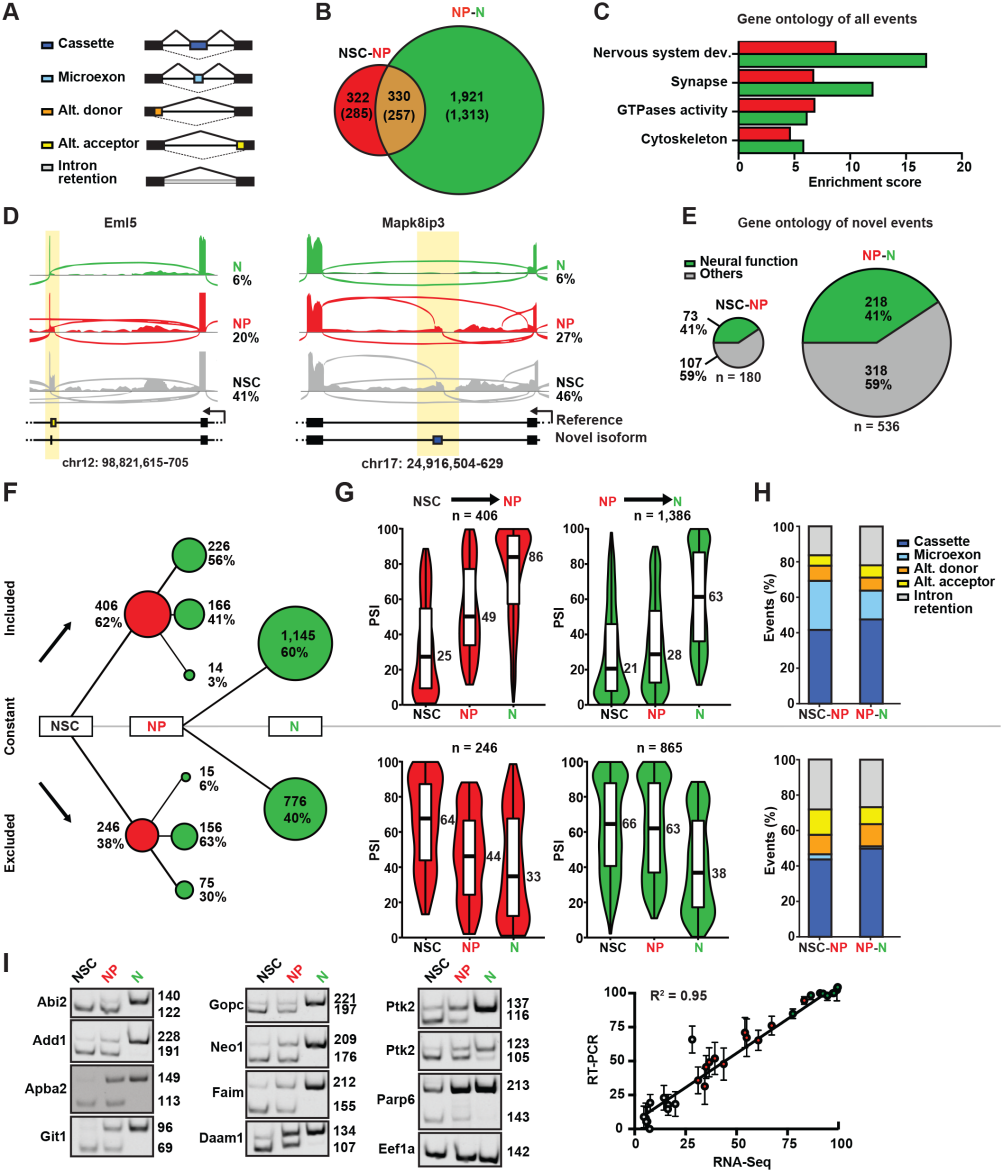
**Assessment of AS during neurogenic commitment.** (A) Classification of types of splicing events into: cassette exons, microexons, alternative donor, alternative acceptor and intron retention. (B,C) Diagrams showing the number of differentially spliced events and corresponding genes (parentheses) (B) and enrichment scores for their gene ontology annotation (C). Analyses were performed for differentially spliced events specifically in the NSC-NP (red), NP-N (green) transitions (B and C), or both (B, overlapping area). (D) Sashimi plots of AS events involving novel transcripts. (E) Proportion of novel transcripts detected in our study to undergo AS and known to regulate nervous system development or function. (F) Patterns of AS classified as events being included, excluded, or unchanged (lines pointing up, down, or flat, respectively) in the progression from NSC to NP and NP to N (red and green circles, respectively). Area of circles are in scale to the number of events. Note the green circles stemming out of the red circles and representing the pattern of AS events changing in consecutive cellular transitions, while AS changing only between NP and N are represented as green circles stemming out of “NP”. (G) Violin plots representing the PSI assessed in NSC, NP and N of events differentially included (top) or excluded (bottom) in the transition from NSC to NP (red, left) or NP to N (green, right). Median PSI values are indicated at the right of each violin plot. Note the gradual and constant changes (left), as opposed to the N-specific, single step change (right), occurring in the NSC-NP versus NP-N transitions. (H) Relative proportion of types (see legend and figure A) of included (top) or excluded (bottom) splicing events. Note the abundance of microexon inclusion (light blue; top) virtually undetected among excluded events (bottom). (I) Validation of inclusion events using primers flanking the alternative exon and giving one exclusion (lower) and one inclusion (higher) qRT-PCR band (left) whose quantification showed a very high correlation coefficient (R^2^=0.95, p<0.0001) (right) when compared to our bioinformatic analysis of combined SRS and LRS transcriptomes of cell types (colors).

Next, we assessed AS characterizing the transition from NSC to NP and N. Quantitative assessment of isoforms abundance is notoriously difficult and unreliable both in the analysis of SRS as well as LRS data (Sarantopoulou et al., 2021). Therefore, we focused our study on classifying splicing events, namely cassette exons, alternative donor or acceptor splice sites and intron retention (Marasco and Kornblihtt, 2023) (Fig. 2A). For this, we used our full transcriptome assembly as a reference while quantifying splicing events based on SRS data only by using Whippet (Sterne-Weiler et al., 2018). Level of inclusion of a splicing event was then expressed as percentage spliced in (PSI), and considered differentially spliced based on the commonly used threshold of ≥10% with a high prediction confidence (see Materials and Methods).

We found a total of 2573 splicing events (1643 genes) changing their inclusion in subsequent stages of the neurogenic lineage, with 652 events (505 genes) differentially spliced during the transition from NSC to NP and 2251 events (1444 genes) from NP to N among which 330 events (257 genes) changed inclusion in both NSC-NP and NP-N transitions (Fig. 2B). We further compared the splicing events found in our study with those reported in VastDB (Tapial et al., 2017) as a reference atlas of RNA-Seq-derived splicing profiles of vertebrate tissues including events not present in the official annotations. Overall, 1946/2573 (75%) events, including novel ones, were also found in VastDB increasing our confidence in the reported novel isoforms and splicing events (data not shown). Gene ontology analysis of all differentially spliced genes, compared to all multi-exonic genes expressed during corticogenesis, highlighted an enrichment in terms related to *neurogenesis* and *nervous system development* in both NSC-NP and NP-N (enrichment scores: 8.7 and 16.8, respectively) with similarly high enrichment scores related to *synapse*, *GTPase* and *cytoskeleton* (Fig. 2C). Several features were detected among these differentially spliced isoforms such as inclusion of novel sequences or use of novel splice junctions (examples in Fig. 2D) with a significant proportion of them (41%) found associated with gene ontology terms related to *neural function* (73/180 genes in NSC-NP and 218/536 genes in NP-N) (Fig. 2E). In turn, these findings highlight the extent of AS within the neurogenic lineage underscoring its potential to regulate corticogenesis to a much greater degree than previously appreciated.

Next, to reveal the dynamics of AS during neurogenic commitment we assessed PSI of variants characterizing NSC, NP and N finding a considerable overrepresentation of inclusion (62%) relative to exclusion (38%) events. This held true in both transitions from NSC to NP (406 versus 246 events) and NP to N (1386 versus 865 events) (Fig. 2F). Moreover, we observed that the overwhelming majority of inclusion/exclusion events in the transition from NSC to NP continued their trend of inclusion/exclusion, or remained constant, in the following transition from NP to N while only a negligible fraction of events showed contrasting patterns, namely included in NP but excluded in N (14 events, 3%) or vice versa (15 events, 6%) (Fig. 2F). In turn, such predominance of inclusion events and bias in splicing patterns during development are in agreement with previous reports not only in the mouse brain (Irimia et al., 2014; Weyn-Vanhentenryck et al., 2018) but also in other organs and species, from chicken to opossum and primates, including humans (Mazin et al., 2021).

Categorizing splicing events according to their patterns of inclusion/exclusion provided a first glance over the trends of AS. To achieve a more comprehensive view, we next expanded our assessment of the number and relative abundance of inclusion/exclusion events (Fig. 2F) to also consider their magnitude, i.e. their change in PSI (Fig. 2G; Fig. S3A). We first started by considering differentially spliced exons in the transition from NSC to NP, irrespectively of their splicing patterns in the subsequent NP to N transition. Among this group, the PSI of exons gaining inclusion (406) constantly increased from a median of 25 in NSC to 49 in NP and 86 in N (Fig. 2G; red, top-left). These values indicate that, overall, events that started to gain inclusion during neurogenic commitment occurred within the least abundant isoforms of NSC and that these isoforms became the most abundant in N. Conversely, exons undergoing exclusion from NSC to NP (246) belonged to the predominant isoforms in NSC that constantly decreased their median PSI from 64 to 44 in NP and 33 in N, ultimately becoming the least-represented isoforms (Fig. 2G; red, bottom-left). Along similar lines, inclusion events in the transition from NP to N (1386) switched from being the minor (PSI of 21 and 28 in NSC and NP, respectively) to become the major (PSI of 63) isoform in N (Fig. 2G; green, top-right). Vice versa, exclusion events (865) switched from the major (PSI of 66 and 63 in NSC and NP, respectively) to the minor (PSI of 38) isoform in N (Fig. 2G; green, bottom-right). Together, this highlighted progressively consistent patterns of exon inclusion/exclusion in consecutive steps of the neuronal lineage. Notably, the choice of splicing pattern did not seem to depend on changes in overall gene expression, as indicated by the lack of correlation between differences in PSI of splicing events and the log2FC of the genes to which they belong (R2≤0.03, Fig. S3B). Moreover, genes harboring inclusion versus exclusion events did rarely overlap (Fig. S3C), although they did not show any significant functional difference (data not shown).

In addition, and extending on previous observations of exon-inclusion during brain development (Mazin et al., 2021; Weyn-Vanhentenryck et al., 2018), we also identified a class of N-specific exons virtually absent in NSC that gained inclusion in NP, and comprising a considerable proportion (112 out of 281 cassette exons) of microexons (Fig. 2H; top), a class of highly conserved events in the nervous system that almost exclusively show inclusion patterns (Irimia et al., 2014; Li et al., 2015b; Mazin et al., 2021; Ustianenko et al., 2017).

To validate these intriguing observations on inclusion patterns, we next selected 11 events showing: (i) low PSI in NSC (<30), (ii) strong PSI gain (≥25) in NP and (iii) high inclusion level in N (>75 PSI) and assessed their abundance by qRT-PCR. This revealed, in all cases, a remarkably high correlation between the bioinformatically predicted PSI and their experimental assessment (R^2^=0.95, *P*<0.0001) (Fig. 2I). Together, these observations reinforce the hypothesis that cell fate specification towards a neurogenic fate involves transcriptome remodeling through AS and, primarily, novel exons inclusion. Most importantly, not only our study highlighted a strong tendency for inclusion events to become the major, if not unique, form in N but also an asymmetry for NP-specific AS patterns to represent a transitory phase prior to the adoption of a definitive, N-specific, AS profile.

### Systematic investigation of protein structural changes resulting from AS

**Fig. 3.**
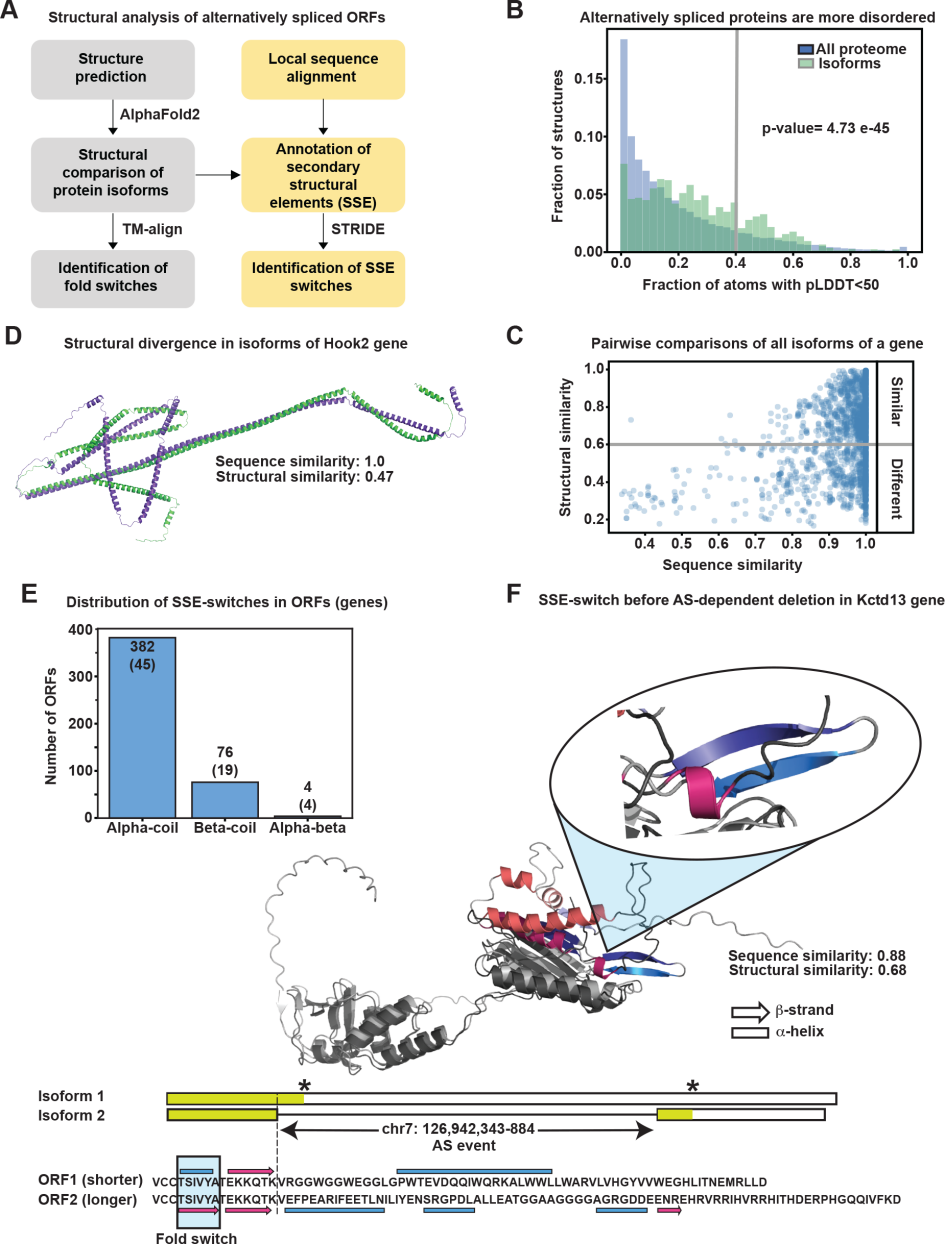
**AS events induce both global and local conformational changes in 3D structures.** (A) Structural analysis of alternatively spliced proteins including global (grey) or local secondary structure element (SSE) switches (yellow). (B) Distribution of the fraction of atoms with pLDDT scores<50 representing disordered structures (threshold indicated by grey bar<0.4; total structures=21,615) in our isoform dataset (green) versus the mouse proteome (blue) (*P*-value=4.73e-45, Kolmogorov–Smirnov test). (C) Pairwise comparisons between all isoforms within one associated gene (TM-score<0.6 threshold, shown as a grey line). (D) Example of global conformational re-arrangement in two isoforms of *Hook2* with high sequence similarity. The change was induced by a small deletion of only two amino acids in a coiled-coil domain. The scores for structural and sequence similarity are shown (scores are normalized by the shorter sequence). (E) Distribution of the secondary structure element switches in ORFs and associated genes (parentheses). Alpha-coil changes are the most common, but beta-coil and alpha-beta changes were also observed. (F) Example of an alpha-beta switch in the gene *Kctd13*, encoding a component of a complex required for synaptic transmission. A fragment of the sequence alignment is shown for the two isoforms and corresponding SSEs depicted as rectangles (alpha-helix) or arrows (beta-strand). The C-terminal parts of the structures are superimposed (grey and dark grey regions), with the SSE switch highlighted (inset) displaying the two beta-strands (blue and violet) and an alpha-helix (purple) despite identical protein sequences. Transcripts structures of the region undergoing AS and the amino acid sequences of the corresponding ORF are depicted below the AlphaFold model. *Kctd13* isoform 1 retains an intron harboring a premature stop codon, resulting in a truncated protein, while isoform 2 continues the translation of the following exon until it encounters another stop codon. Translated regions are indicated as colored in the transcripts structure, while non-translated ones are left white. Stop codons are depicted with asterisks. The residues common to the two resulting ORFs are indicated in bold. Combining short- and long-read sequencing with AlphaFold analysis of protein conformational changes reveals the extent and potential biological significance of AS in neurogenic commitment during mouse embryonic brain development.

To investigate how AS impacts protein structure and, potentially, function we predicted the conformational changes of those isoforms switching during neurogenic commitment using AlphaFold2 (Jumper et al., 2021). Modeling of 3D structures and prediction of their global and local conformational changes (Fig. 3A; left) was limited to protein isoforms of up to 800 amino acids, hence, compromising between the computational time needed to model the structures and the quality of the retrieved models. This resulted in the selection of 212 genes of which more than half (127) were associated with GO terms related to organ development, macromolecular complex assembly and protein localization/transport and one third (71) with brain development specifically (not shown). Next, we extracted the 3D structures of the corresponding 987 isoforms undergoing AS during the transitions from NSC to NP and N.

In agreement with previous studies (Hegyi et al., 2011; Romero et al., 2006), we observed that the structures in our set of isoforms were significantly more disordered than the average mouse protein (21,615 proteins from canonical proteome) when using the generally accepted percent threshold of residues with predicted local distance difference test (pLDDT score<50) to define disorder (Fig. 3B; gray line). Next, and considering the difficulties in the structural comparison of isoforms with large disordered regions, we limited our analysis to structures where the fraction of residues with very low prediction confidence score (pLDDT score<50) did not exceed 40%, and compared the sequence and structural similarity of all isoforms associated with a single gene.

Not surprisingly, we observed that the template modeling (TM) scores indicative of structural similarity generally increased with increasing sequence similarity (Fig. 3C). However, a remarkably high proportion of genes (78 out of 212; or 37%) mainly with a high sequence similarity (median=98%) were found to result in isoforms with substantially different structural conformations (TM-score<0.6) (Fig. 3C; bottom right). As one example among this group of genes, two *Hook2* isoforms only differed by as little as two amino acids (nearly 100% sequence similarity), yet revealed a remarkably different orientation of their N-terminal helical packing (47% structure similarity) (Fig. 3D). Notably, the protein domain essential for Hook2 function to bind microtubules resides within this helical packing, underlying the potential of even a negligible AS switch by two ammino acids to result in a conceivable altered function (Fig. S4A).

While TM-scores are informative of large conformational changes and global structural similarities, we next investigated whether AS had the potential to also introduce more subtle, local changes in specific secondary structural elements (SSE). To assess this, we combined SSE annotation with local sequence alignment (Fig. 3A; right) finding primarily alpha- and beta-coil switches and, more rarely, alpha-beta switches (Fig. 3E). Such short sequences that can adopt both alpha and beta conformations depending on the structural and sequence context within which they are embedded are known as chameleon sequences (Li et al., 2015a). In our set of genes, we found that these chameleon transitions could be induced just by the presence or absence of a distant spliced region with the rest of the structural and sequence neighborhood context of the protein being nearly identical. A notable example of an alpha-beta switch was found in the gene *Kctd13* (Fig. 3F; Fig. S4B), encoding a compo­­nent of a complex required for synaptic transmission (Escamilla et al., 2017) and implicated in neurodevelopmental disorders such as macrocephaly (Golzio et al., 2012) [although a subsequent study could not confirm the association with this latter phenotype (Escamilla et al., 2017)]. This example highlights how identical sequences can adopt different structural conformations as a result of an AS event occurring 7 amino acids away.

PLDDT scores, considered as one of the reliability measures of the structural models (Jumper et al., 2021), were used to estimate confidence and flexibility in the switch regions (Fig. S4B). Interestingly, the most intriguing cases of switches, the alpha-beta ones, exhibited high pLDDT scores within the structure-switching regions, with three out of the top four models presenting a median score above 80 (Fig. S4C, right panel). Notably, alpha-coil switches present lower pLDDT values than other switches (median ∼50) (Fig. S4C, left). It was shown that pLDDT scores are highly correlated with different measures of protein disorder (Binder et al., 2022; Zhao et al., 2023), namely low pLDDT scores mean high structural disorder and flexibility. The relatively low pLDDT scores observed for the alpha-coil switches could therefore be explained by an increased level of disorder in these regions (e.g. loops), potentially providing the necessary flexibility for the proteins to perform their function.

Several other examples of both global and local conformational changes resulting from AS events occurring within or outside the affected protein domain were found in our study (data not shown). Future studies are needed to investigate how many of them can lead to protein functional diversification relevant for cell fate commitment during brain development.

## DISCUSSION

Our study explores a new avenue for assessing gene function in cell fate commitment by looking at the potential diversification of protein structure resulting from AS rather than, as universally adopted, measuring gene expression alone. As a traditionally poorly investigated aspect of cell biology, a comprehensive assessment of AS required the advent of new sequencing technologies and bioinformatic tools. Similarly, reliable prediction of protein structures from their sequence alone required the implementation of machine learning to interrogate depository databases of crystal structures. Here, we combined these tools starting with a validated reporter mouse model allowing the identification of cell populations recapitulating neurogenic commitment during brain development.

In doing so, our work advances the field in several contexts. First, it exploits a still poorly employed methodological approach to assess AS by the combination of SRS and LRS. Integrating full-length transcripts identified by LRS with those assembled with SRS data has been instrumental for identifying novel isoforms in model organisms (Li et al., 2022; Shumate et al., 2022), as well as providing annotations for poorly studied organisms (Lin et al., 2023). Here we adopted a similar strategy to assess cell-type-specific AS dynamics and further extend this to predict its effects on protein structure by machine learning based protein structure determination. Our study also provides the field with a new resource of cell-specific transcriptome annotations of the developing mouse brain including nearly 50,000 novel isoforms and 2500 previously unknown splicing events. Third, it extends previous reports on the significance of AS in the context of neuronal maturation (Liu et al., 2018; Zhang et al., 2016) to also reveal its full extent and potential significance in cell fate commitment of stem and progenitor cells. Several aspects of our study are worth discussing toward better understanding the biological significance of our observations.

In agreement with previous assessments of AS profiles during organogenesis (Mazin et al., 2021), and likely a result of differential expression levels of splicing factors (not shown), our analysis in the context of cortical development revealed patterns of AS events strongly skewed towards inclusion of exons in both transitions from NSC to NP and NP to N. Most importantly, the magnitude of the observed inclusion events was such that, on average, isoforms only marginally represented in NSC, if any, became the main, if not unique, isoform in N with NP representing a transient intermediate cellular state between the two. In essence, AS alone was revealed to have a much greater impact in remodeling the transcriptome profile from NSC to N than previously thought and independently from changes in gene expression.

Also expanding on previous findings on a peculiar class of short exons in the nervous system (Irimia et al., 2014; Li et al., 2015b; Mazin et al., 2021; Ustianenko et al., 2017), we found that virtually all alternatively spliced microexons were progressively more included as development proceeded from NSC to N and accounting for more than one third (40%) of included cassette exons during the step of neurogenic commitment. This observation strengthens the hypothesis that this class of highly conserved exons might have a central role in redefining neuronal identity highlighting the importance in our study of assessing specific cell sub-populations to reveal the full potential of AS patterns in fate commitment.

Next, we investigated the impact of AS on protein structure by AlphaFold2 and found a high proportion of disordered regions in our set of alternatively spliced genes. In fact, AS often affects intrinsically disordered regions (Li et al., 2015a) and tends to avoid globular domains or affect them only marginally at locations where the exposed hydrophobic surface is minimal (Hegyi et al., 2011). An emerging concept in the field is that differential inclusion of disordered segments can favor new protein interactions and, hence, change the context in which the molecular function of the protein is carried out (Buljan et al., 2013). Our observation of an increase in disordered isoforms arising concomitantly with neurogenic commitment could be interpreted as a mechanism to remodel the interactome during cell-fate commitment.

Finally, it is important to bear in mind that while AlphaFold2 has remarkable accuracy in predicting ordered proteins conserved across evolution, its accuracy significantly decreases when predicting disordered regions and sequences with only few homologs. Applying stringent quality filters and limiting our analysis to the comparison of 3D conformations with less or equal to 40% disordered regions reduced, but certainly did not remove, noise in predicting conformations due to uncertainty. Still, while false results are difficult to quantify, the extent and number of structural changes observed between isoforms that were nearly identical in sequence appears to be far greater than what prediction errors may justify. This was similarly the case both when the structural change occurred within the AS event and, more remarkably, when the event was far away in sequence. In addition, we found several chameleon sequences that adopted different secondary structures in specific cell types as a result of AS. While these regions are long known to exist, their structural switch was assumed to be dependent on substantial changes in their structural and sequence contexts (Gendoo and Harrison, 2011; Li et al., 2015a) as opposed to, as observed in our study, being triggered by small perturbations within nearly identical sequence contexts.

While more studies are needed to validate the predicted structural changes, their impact on protein function and, most importantly, their relevance in brain development, we hope that our study may provide a new resource and conceptual framework to better address the significance of AS during cell fate commitment and organogenesis.

## MATERIALS AND METHODS

### SRS and LRS transcriptome assembly of NSC, NP and N

A previous SRS dataset of FAC-sorted *Btg2*::RFP/*Tubb3*::GFP cells of the E14.5 mouse cortex obtained in three biological replicates (Aprea et al., 2015) was used to generate a new transcriptome assembly identifying NSC, NP and N. Paired-end reads were mapped to the Ensembl (GRCm38.p6) mouse genome using Hisat2 (Kim et al., 2019) and Stringtie used to assemble the mapped reads into transcripts using Ensembl as a reference (only transcripts with support level 1/2) (Pertea et al., 2015). Only transcripts with >10 supporting reads were included and fusion transcripts removed, resulting in 25,710 transcripts. Reference transcriptome was not included in the final assembly. For the generation of a new LRS dataset, Isoseq library preparation was performed on 300 ng of RNA (RIN ≥9.3) of each cell type (isolated as described above by three biological replicates pulled together) using NEBNext^®^ Single Cell/Low Input cDNA Synthesis & Amplification (NEB), Iso-Seq Express Oligo Kit (PacBio) and SMRTbell Express Template Prep Kit 2.0 (PacBio). Samples RNAs were barcoded, retrotranscribed into cDNA and multiplexed before sequencing in a single run using PacBio Sequel II 8M SMRT cell (version 2.1) (Fig. S1A). The raw data were processed with Isoseq3 workflow (≥3 passes and accuracy≥0.9 for CCS reads and≥7 passes for high-quality transcripts), resulting in 3,577,360 high-fidelity (HiFi) reads with an average length of 3437 bp. Importantly, an equivalent number of raw reads of the three cell populations was achieved (1,071,261 reads for NSC, 994,801 for NP, and 1,304,212 for N). Next, the HiFi reads were processed with the Isoseq3 workflow, generating a total of 3,343,340 full-length non-chimeric (FLNC) reads with a mean length of 3333 bp. The FLNC reads were further clustered into 213,010 high-quality transcripts with a minimum mapped concordance of 95%, minimum mapped coverage of 99%, and a minimum of seven passes. Only 898 transcripts were classified as low-quality transcripts and they were not used in further analyses (Fig. S1A). High-quality transcripts were aligned to *Mus musculus* GRCm38/mm10, collapsed to non-redundant transcripts, and 5′ degraded transcripts and potential artifacts removed using either pipeline 1 [including gmap (Wu and Watanabe, 2005), cDNA_Cupcake (https://github.com/Magdoll/cDNA_Cupcake) and SQANTI3 (Tardaguila et al., 2018)] or pipeline 2 [including deSALT (Liu et al., 2019), Tama (Kuo et al., 2020) and SQANTI3] with standard parameters, resulting in 76,077 and 80,782 transcripts, respectively. After removal of fusion transcripts, 75,275 transcripts from pipelines 1 and 78,242 from pipeline 2 were merged into a LRS transcriptome assembly with Tama merge. A tolerance of 25 nt for the 5′ ends (-a 25 parameter) and a threshold of 10 bp for splice junctions (-z 10) and 3′ ends (-m 10) were applied for merging transcripts, resulting in several transcripts from one pipeline to merge with more than one transcript from the other pipeline, and vice versa (see schematic in Fig. S1B). Overall, 70,573 and 71,682 transcripts from pipeline 1 and 2, respectively, were merged into 70,290 common transcripts, and 4,706 and 6,671 into 4,698 and 6,446 transcripts specific to pipeline 1 and 2, respectively, for a total of 81,434 LRS transcripts. SQANTI3 was used for classification of transcripts by comparing it with the best-matching reference transcript from Ensembl (GRCm38.p6, v101), NCBI_RefSeq (https://hgdownload-test.gi.ucsc.edu/goldenPath/mm10/bigZips/genes/), and Gencode (vM10). Transcripts were considered known when containing only known splice as either all (full-splice match) or only part (incomplete splice-match) of the best-matching reference. Transcripts were classified as novel in catalog (or not in catalog) if containing a novel combination of known donor and acceptor sites (or novel donor and/or acceptor site). The transcription start (TSS) and end (TTS) sites of all transcripts were compared with annotated TSS and TTS. Following standard practice in the field, a TSS or TTS was considered to be supported when located within 100bp upstream or downstream of an annotated TSS/Cage peak or an annotated TTS site/PolyA motif. A high percentage of reported transcripts was supported by the annotated sites (76,820/81,434, 94% with TSS support; 78,119/81,434, 96% with TTS support). Noteworthy, we observed that the majority of novel transcripts start (NNC start 90%; NIC start 97%) and end (NNC end 98%; NIC end 98%) was supported by the reference (Fig. S1C, left). Reliability of novel splice sites and novel combinations of known splice sites was checked by their support by SRS reads. Only 16.5 and 7.6% novel splice sites and novel combinations of known splice sites, respectively, were not supported by any SRS reads, while about half of them (47.7 and 55.6%, correspondingly) were supported by at least 10 short-reads (Fig. S1C, right). Novel splice sites and novel combination of splice sites were kept only if supported by short reads or if classified as canonical splice site. Finally, LRS and SRS transcriptome were merged in a unique assembly with Tama merge and classified with SQANTI3 as previously described. The final assembly resulted in 97,240 transcripts from 14,473 genes, of which 9310 common transcripts (derived by 9647 and 9205 transcripts from SRS and LRS, respectively) and 15,703 (from 16,070 initial transcripts) unique to SRS and 72,227 (from 72,229 initial transcripts) unique to LRS (Fig. S1A). SQANTI3 was used to classify isoforms in the final transcriptome as known (FSM, PSM), novel (NIC, NNC) or others (intergenic, antisense, etc.). The proportion of transcripts presenting RT-switching, non-canonical splice sites or predicted to undergo NMD was also evaluated with SQANTI3 (Fig. S1D, left). Of these, a total of 25,592 were classified as NIC, with 3,177 and 21,530 supported only by SRS and LRS, correspondingly, and 885 by both assemblies. Transcripts NNC amounted to a total of 13,496, of which 2,704 from SRS transcriptome, 10,485 from LRS transcriptome and 307 from both transcriptomes (Fig. S2). All experimental procedures were performed according to local regulations and approved by the “Landesdirektion Sachsen” under the licenses 11-1-2011-41 and TVV 16/2018.

### Detection of splicing events

Paired-end reads (Aprea et al., 2015) were mapped to the mouse genome annotated with the developing cortex transcriptome assembly using STAR (Dobin et al., 2013). Whippet (Sterne-Weiler et al., 2018) was then used to estimate exon and intron inclusion in each replicate. Lowly expressed genes (<0.5 transcripts per million) were excluded from analysis. AS events were defined as absolute PSI change ≥10 with a minimum probability of 0.9 and a confidence interval width ≤0.3. When the exon boundaries did not coincide among a gene's transcripts (alternative 5′ or 3′ splice site choice), Whippet divide the exon into nodes and calculate a PSI for each of them. Contiguous nodes showing similar PSI levels and patterns and likely belonging to a single exon were fused, their PSI averaged and considered a single event. The cluster module of DAVID (Huang et al., 2009; Sherman et al., 2022) was used for clustering and enrichment evaluation of gene ontology terms of differentially spliced genes. Only GO terms with a minimum enrichment score of 3 were considered and aggregated together when redundant. For each group the enrichment score of the highest GO term was reported. Multiexonic genes expressed with a minimum of 0.5 tpm in the cell populations of interest were used as a background. Only genes that could be assigned to an Ensembl annotated gene were used in this analysis.

### Identification and validation of AS isoforms and novel splicing events

Isoforms with high abundance (>4 unique splice-junction reads across all splice-junctions) were considered and AS events coordinates compared to the transcripts annotated in our dataset. AS events coordinates were compared with transcripts structures in order to identify isoforms with (inclusion isoforms) and without (exclusion isoforms) the events. Only AS events assigned to at least one inclusion and one exclusion isoform, in either our annotation or official ones, were considered for further analysis. AS events and exclusion sites predicted to have a novel acceptor and/or donor site or a new combination of known splice sites were identified by SQANTI3, while the AS events containing novel sequences were identified by bedtools. SQANTI3 classification of inclusion and exclusion isoforms as non-sense mediated decay (NMD) was used as a proxy to assess the propensity of an AS events to elicit NMD. AS events were considered to induce NMD when included/excluded if all the inclusion/exclusion isoforms detected resulted in NMD transcripts. If at least one coding inclusion and one coding exclusion isoforms was identified, the AS event was not considered to induce NMD.

For novel isoforms and AS event validation, RNA with RIN>8.0 from C57BL/6J lateral cortices was collected and qRT-PCR performed to assess novel splice junctions, as well as levels of inclusion changes of AS events. For each target sequence, amplification by PCR was carried out with 1 ng of cDNA with standard conditions: 98°C 30 s, 27-33 cycles of 98°C 15 s, 68°C 25 s, 72°C 30 s, 72°C 2 min. PCR products were resolved in 15% polyacrylamide gels.

### Structural analysis

AlphaFold2 was used to model the 3D structures of isoforms (Jumper et al., 2021). For each isoform sequence, AlphaFold2 was used with default parameters, installed on a local machine, with the use of three GPUs. The best-ranked model (ranked by AlphaFold2) was chosen out of five models provided. To access global structural rearrangements between isoforms, global structural alignment was performed between each pair of isoforms within each gene using the TM-align algorithm (Zhang and Skolnick, 2005) computing the TM-score for each alignment and allowing the assessment of structural similarity between proteins. To identify local structural rearrangements, a pipeline was developed (Fig. 3A) including protein local sequence alignment by the Smith-Waterman algorithm (python package Biopython). The computed AlphaFold structural models were annotated with secondary structure elements (SSE) by STRIDE (Heinig and Frishman, 2004). SSE switching regions of isoforms were defined when changes in the assignment of SSE for the corresponding regions in protein sequence (identified by alignment) were observed for ≥5 consecutive amino acids. Figures were generated by Pymol, Jalview and Python matplotlib.

### Code availability

All codes regarding transcriptome assembly, AS detection, processing, and analysis of AlphaFold2 model structures can be found in the dedicated github page: https://github.com/BeatrizToledo/paper

## Supplementary Material

10.1242/biolopen.061721_sup1 Supplementary information
