# Supplementary material for: Characterization of alternative splicing during mammalian brain development reveals the extent of isoform diversity and potential effects on protein structural changes
Source: Biol Open. 2024 Oct 10;13(10):bio061721. doi: 10.1242/bio.061721 (PMC11554263; doi:10.1242/bio.061721)
Supplement: Supplementary information [file biolopen-13-061721-s1.pdf]

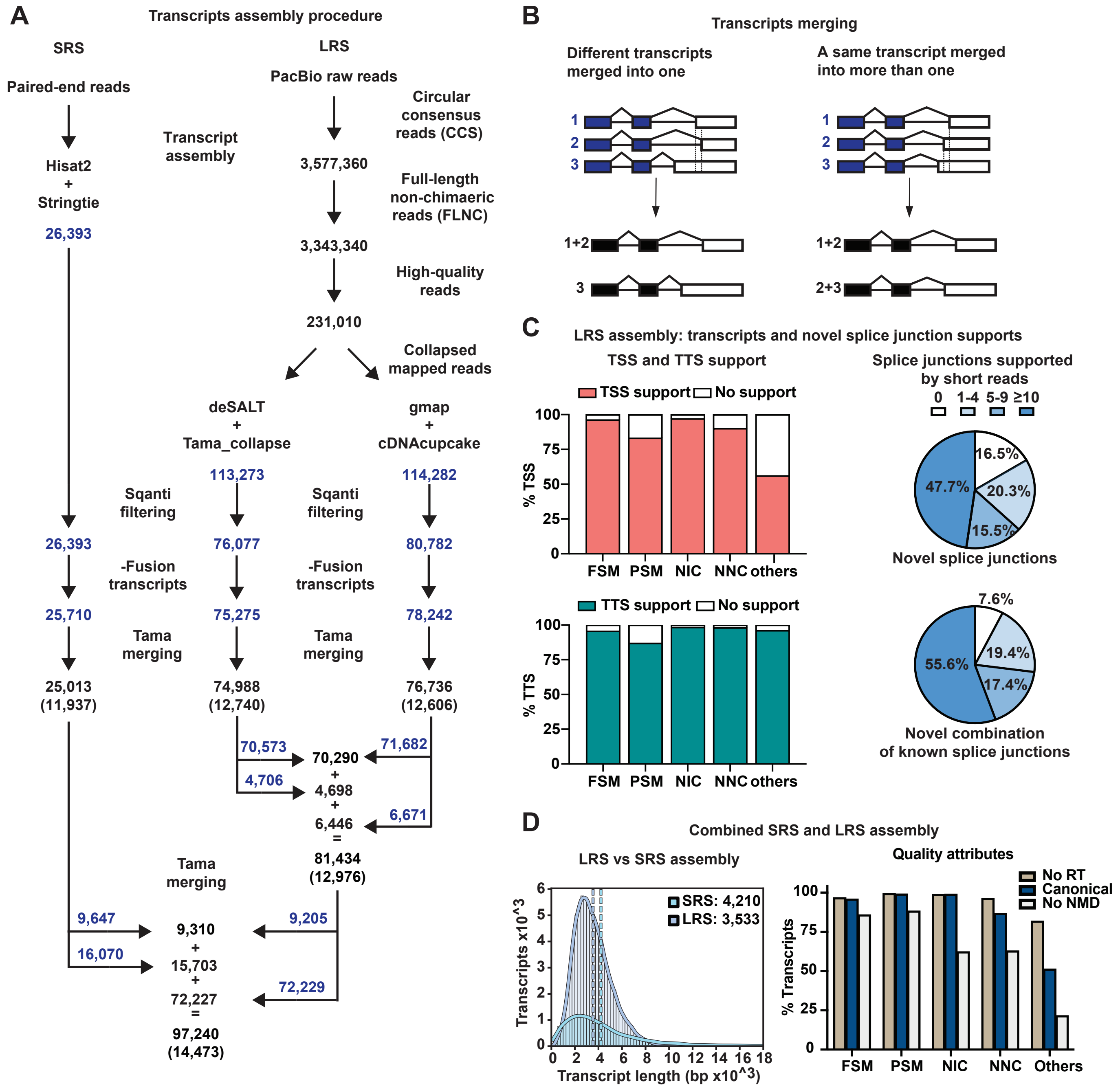


Biology Open • Supplementary information

**Fig. S1. Long-read sequencing and transcriptome assembly. A)** Schematic of sequencing strategy and transcriptome assembly with short- (left) and long (right) reads. **B)** Processing of short and long-reads with different pipelines to collapse and merge reads into non-redundant isoforms. The merging process can associate one isoform from one pipeline to several of another pipeline and vice-versa. **C)** Transcripts support data on their transcription start and end sites (TSS and TTS, respectively), as well on novel junctions and combination of known junctions are shown for isoforms assembled with LRS data. **D)** Left: Length distribution of isoforms assembled with SRS or LRS; right: quality attributes of isoforms of the combined SRS and LRS assembly, showing the percentage of isoforms displaying all canonical junctions, non-RT switching artifacts and predicted to generate a protein isoform (i.e., not-undergoing non-sense-mediated decay, NMD).

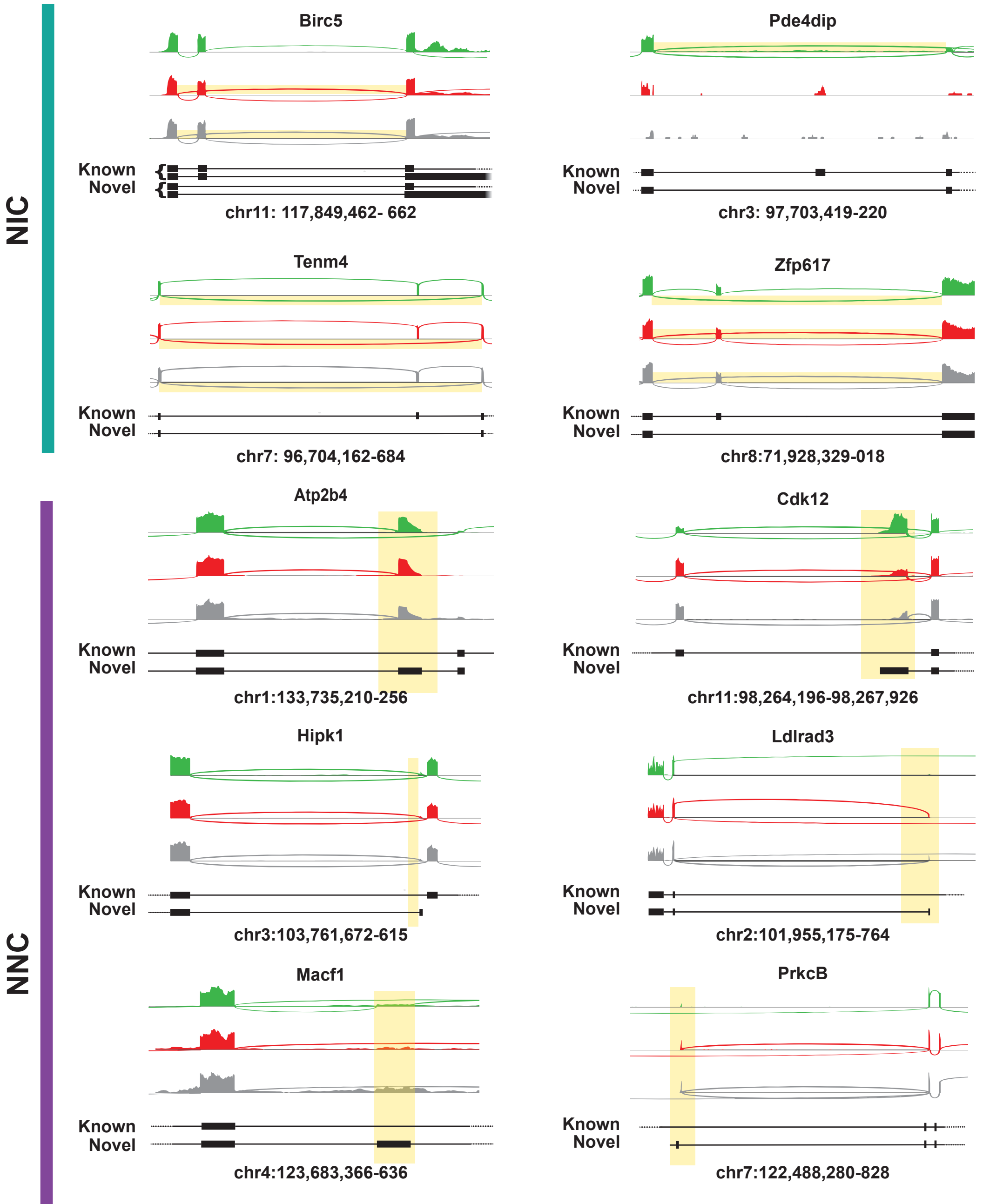


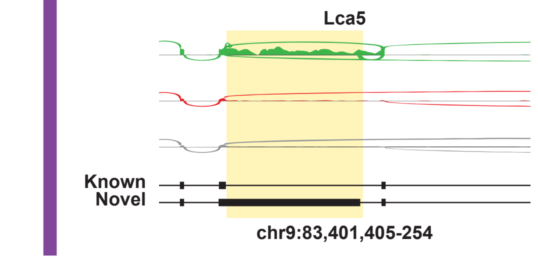


| | Only SRS | Only LRS | SRS and LRS |
|---|---|---|---|
| NIC | 3,177 | 21,530 | 885 |
| NNC | 2,704 | 10,485 | 307 |

**Fig. S2. Novel isoforms** Transcripts novel in catalog (NIC) and novel not in catalog (NNC) derived from SRS, LRS or both (table) and sashimi plots showing sequencing data of events selected for validation relative to Fig. 1C. Novel junctions or exons are highlighted with a yellow box.

Biology Open • Supplementary information

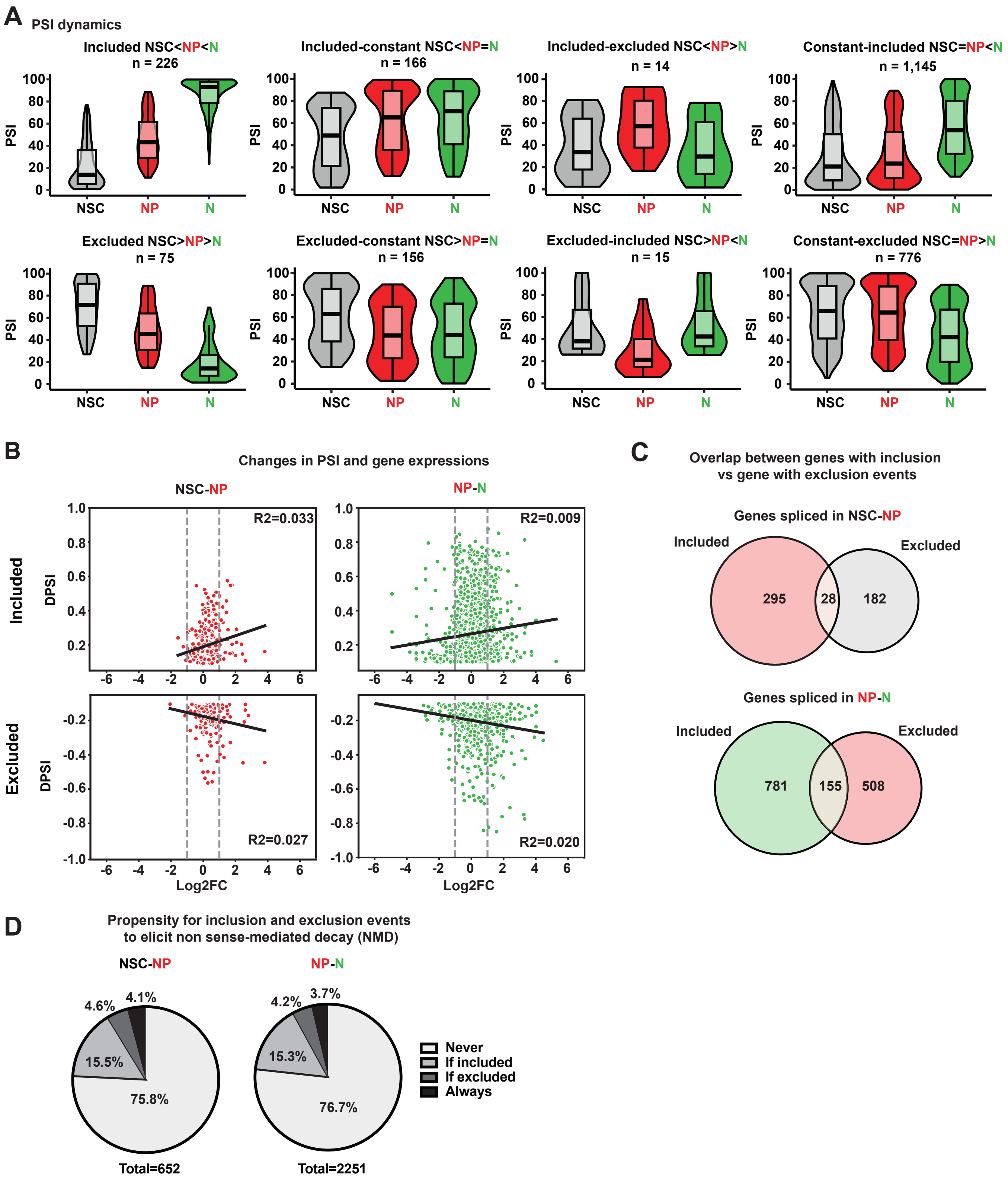


Biology Open • Supplementary information

**Fig. S3. AS events PSI dynamics and expression of their relative genes. A)** Violin plots representing the PSI assessed in NSC, NP and N of events showing any possible pattern of inclusion/exclusion. **B)** Correlation between gene expression and PSI of relative AS events increasing (top) or decreasing (bottom) their PSI in NSC-to-NP (red) and NP-to-N (green) transition. No significant relationship could be found between changes in PSI (DPSI) and the expression of the corresponding alternatively spliced gene. **C)** Venn-diagram showing the overlap between genes presenting exclusion and inclusion AS events in NSC-to-NP and NP-to-N transition. Genes that present both inclusion and exclusion events in the same transition are a minority, while most genes present either inclusion or exclusion AS events. **D)** Pie-chart of the proportion of isoform predicted to undergo NMD when an AS is included/excluded. The majority of isoforms are predicted to give a protein regardless of the differential inclusion of the AS events detected.

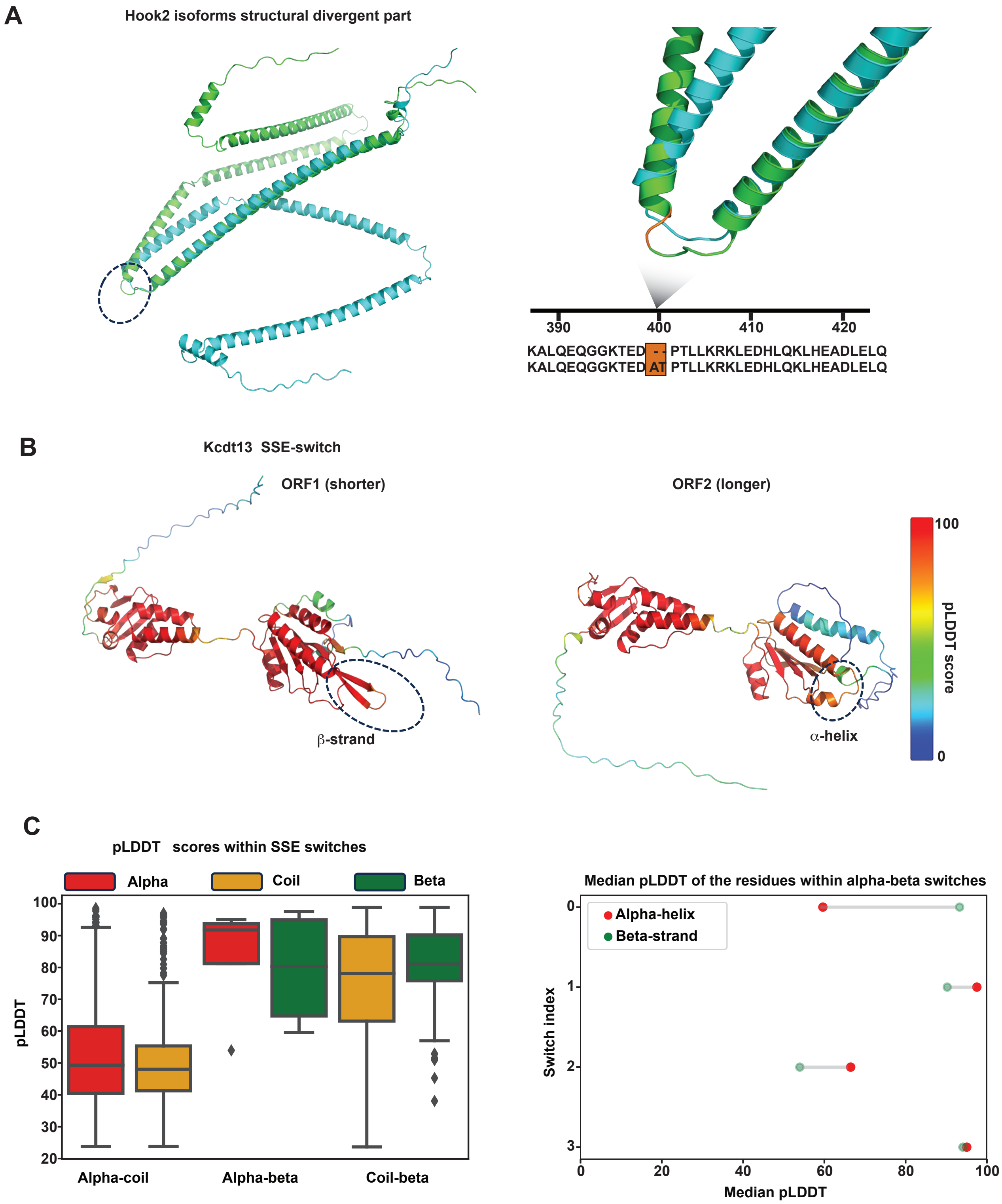
A
Hook2 isoforms structural divergent part
390
400
410
420
KALQEQGGKTED - - PTLLKRKLEDHLQKLHEADLELQ
KALQEQGGKTED AT PTLLKRKLEDHLQKLHEADLELQ
B
Kcdt13 SSE-switch
ORF1 (shorter)
ORF2 (longer)
β-strand
α-helix
100
pLDDT score
0
C
pLDDT scores within SSE switches
Alpha
Coil
Beta
pLDDT
Alpha-coil
Alpha-beta
Coil-beta
Median pLDDT of the residues within alpha-beta switches
Alpha-helix
Beta-strand
Switch index
Median pLDDT

**Fig. S4. AlphaFold2 prediction of isoform structural differences A)** Alignment of parts of the structures in the example of global conformational re-arrangement in isoforms (in cyan and green) of *Hook2* (ENSMUSG00000052566). The first 300 residues were removed from each isoform to provide the alignment of the structurally divergent part. The difference between two isoforms in the alignment (deletion of two amino acids, shown in orange) is shown on the right panel. **B)** Structures of isoforms in the example of the switch before deletion in *Kctd13* (ENSMUSG00000030685). Structures are colored according to pLDDT scores provided by AlphaFold2, where red regions correspond to the prediction with high reliability, and blue to low. The areas with switch are shown with dashed circles. **C)** Box plots showing the distribution of pLDDT scores for the SSE switches, grouped by SSE. Each box is delimited by the first quartile (Q1 or 25%) and the third quartile (Q3 or 75%), with the median (Q2 or 50%) represented as a line within the box. "Whiskers" extend to points within 1.5 times the interquartile range (IQR) from the lower and upper quartiles. Observations outside this range are considered outliers and are displayed as dots. Right: median of pLDDT scores of the regions displaying alpha-beta switches.

Biology Open • Supplementary information
